# Implicit Bias in Counseling for Permanent Contraception: Historical Context and Recommendations for Counseling

**DOI:** 10.1089/heq.2020.0025

**Published:** 2020-07-17

**Authors:** Cosette A. Kathawa, Kavita Shah Arora

**Affiliations:** ^1^ School of Medicine, Case Western Reserve University, Cleveland, Ohio, USA.; ^2^ Department of Obstetrics and Gynecology, MetroHealth Medical Center, Cleveland, Ohio, USA.

**Keywords:** sterilization, contraceptive counseling, implicit bias, contraception, ethics, permanent contraception

## Abstract

We provide an overview of the causes, manifestations, and potential mitigating steps regarding implicit bias in counseling for permanent contraception. The historical context of sterilization abuses and the implications of these on society's notions of fitness for parenthood are reviewed. We present contemporary examples of contraceptive coercion and discuss the impact of implicit bias from health care providers. Finally, we outline steps for ensuring a patient-centered shared decision-making ethical approach to permanent contraceptive counseling.

## Introduction

In the United States, conversations surrounding contraceptive choice and permanent contraception are complicated by the historical backdrop of state-sanctioned eugenics programs meant to control the reproduction of women deemed unfit to parent. Stratified reproduction,^1,2^ in which the fertility of some women was valued and that of others was devalued, is a clear example of structural racism and sexism. Contemporary ramifications of these policies remain, including the Medicaid Consent to Sterilization form, which requires a waiting period to reduce the potential for coercion and ensure informed consent. Although ample evidence exists^3^ that this policy may be a barrier to care for the populations it was designed to protect, simply eliminating it altogether seems problematic as present-day examples of ongoing bias and discrimination are unfortunately available. For example, >100 women in California prisons were coercively sterilized between 1997 and 2010.^4^ In 2017, a judge in Tennessee offered incarcerated persons reduced sentences for receiving permanent contraception or long-acting reversible contraception (LARC), such as intrauterine devices or contraceptive implants.^5^ Such actions are clearly unjust and have been investigated and formally reprimanded.

Unfortunately, the same ideas that informed these coercive policies continue to shape our biases today. In contrast to these overtly discriminatory policies that exemplify the intersectional problem of gendered racism,^6^ implicit bias is more difficult to identify and regulate. Implicit bias is the application of unconscious attitudes informed by a person's background and experiences about particular social groups. Although we all have biases influencing our perceptions and actions, these biases can lead to problematic assumptions about people based on the social groups to which they belong. Health care providers are not immune to such bias, and display the same degree of implicit bias as the general population. These biases can influence clinical decision-making.^7^ On the individual level, such bias is associated with poor communication during visits and poor satisfaction for minority patients.^8^ In a study where providers were presented with clinical vignettes that were identical except for patient characteristics such as race or gender, providers had differing interpretations of the clinical scenarios, potentially due to bias.^9^ On the aggregate public health level, counties in the United States with the highest levels of racial bias have greater racial disparities in birth outcomes.^10^

Implicit bias has also been shown to seep into and impact contraceptive outcomes. Low-income women and black and Latina women are more likely to report discriminatory practices in contraceptive counseling, including being advised to limit childbearing, compared with middle-class white women.^11,12^ Such bias can manifest clinically as favoring certain contraceptive methods solely due to efficacy, counseling patients belonging to particular demographic groups differently, providing unbalanced information, or minimizing side effects.^13^ One study of black and Latina women's experiences showed perceived bias in counseling and pressure to accept particular methods despite not aligning with the patient's reproductive goals. In this study, participants accepted the recommended method, but quickly discontinued it. These experiences engendered distrust of providers and hesitancy surrounding future contraceptive use.^14^ In another study, providers were more likely to recommend intrauterine devices to black women regardless of socioeconomic status as well as to Latina women of low socioeconomic status.^15^ Yet, as a result of historical reproductive coercion, patients may distrust LARC and may view the uneven promotion of LARC compared with other methods with suspicion.^16^

Although such experiences contrast with the earlier examples of overt bias and structural racism, implicit bias serves as an insidious barrier to true reproductive choice and justice. Ethically, such bias compromises patient autonomy, informed consent, and the justice and beneficence principles to treat patients fairly and act in their best interest. Furthermore, implicit bias in contraceptive counseling undermines the principle of nonmaleficence, as patients may be harmed by discontinuation of an undesired contraceptive method and by the long-term impact on their willingness to interact with the health care system.

## Permanent Contraception

Within the realm of contraceptive care, implicit bias impacting access and fulfillment to permanent contraception is especially problematic given the invasive and permanent nature of these contraceptive methods. Specifically, bias in counseling for permanent contraception can lead to ramifications for patients given the difficulty of reversing these procedures and unintended pregnancies resulting from unfulfilled requests. For permanent contraceptive counseling in particular, implicit bias manifests in two distinct manners—(1) counseling patients with certain demographic and clinical characteristics toward permanent contraception and (2) counseling patients with other characteristics away from permanent contraception.

Offering permanent contraception for those patients in whom pregnancy poses dramatic health risks is both ethical and high-quality care. Furthermore, clinicians have a responsibility to take steps within their own practices and advocate for systemic change to remove external barriers to desired permanent contraception, such as mergers with religiously affiliated hospitals^17^ and the Medicaid sterilization policy.^3^

Variations in sterilization rates by demographic and clinical characteristics such as age, race, and parity are well documented in the literature.^18,19^ For example, women of color request permanent contraception more often than white women.^20^ Fulfilling a patient's autonomous preference for permanent contraception is important; however, recommendations toward these procedures based on demographic and clinical characteristics due to subconscious notions of what a “good mother” looks like are unethical. In one study, physicians were presented with clinical vignettes of patients seeking tubal sterilization that differed by various demographic factors. These physicians were most willing to sterilize older, postpartum, parous, black, or poor women.^21^ In another survey, obstetrician-gynecologists recommended sterilization to women not initially requesting sterilization on the basis of appropriate medical reasons such as medical history, preterm birth, and surgical history. However, demographic factors such as age and parity, as well as social factors such as the partner's agreement, insurance, religion, education, and race were also reported as reasons to recommend sterilization.^22^ In the absence of a compelling medical rationale, permanent contraceptive counseling should be nondirective and clinicians should examine their biases before counseling patients toward permanent contraception.

Similarly, clinicians should avoid directive counseling against permanent contraception. In the survey mentioned earlier, obstetrician-gynecologists declined to perform permanent contraceptive procedures in women requesting the procedure due to age, parity, risk of regret, their partner's disagreement, and education.^21^ Although difficult to assess in a survey-based study, these findings may represent a paternalistic overriding of patient autonomy. For this reason, the Ethics Committee of the American College of Obstetricians and Gynecologists advises that imposing arbitrary thresholds for age, parity, gestational age at delivery, and so on is inappropriate.^23^ Although patients should be informed about the risk of regret, it is important to be aware of the flaws in this body of literature. The available data regarding poststerilization regret may represent the conflation of factors such as misunderstanding the permanence of sterilization; coercion from the patient's partner, family, or clinician; restriction of choice or personal history of unreliable contraceptive use due to structural barriers to care; and imperfect research methodology in assessing regret.^23^

Clinicians should also be cautious of statistical discrimination, in which clinicians erroneously utilize epidemiological data or anecdotal experience to guide their recommendations without considering patients' unique needs and desires.^15,24^ For instance, higher rates of unintended pregnancy in women of color should not influence clinicians' likelihood of counseling toward permanent contraception, and higher rates of so-called poststerilization regret in young women should not lead clinicians to automatically discourage permanent contraceptive methods in these patients.

## Patient-Centered Counseling

Permanent contraceptive—and more broadly, all contraceptive—counseling should be rooted in a patient-centered shared decision-making approach (Table 1). This approach aligns with the principles of reproductive justice, a framework created by black scholars and activists that acknowledges the right not to have a child, the right to have a child, and the right to parent children in safe and healthy environments.^25^ Awareness of the ways in which implicit bias influences contraceptive counseling is critical in ensuring genuine reproductive autonomy and justice. Patients' chosen method of contraception may reflect personal, familial, and cultural factors. For example, women will weigh effectiveness differently relative to other characteristics, such as ease of use, privacy, cost, impact on menstrual cycles, reversibility, and lack of hormones.^26^ By using a standardized process of eliciting patient values, clinicians can minimize the impact of their own implicit biases. Clinicians should also elicit patients' contraceptive knowledge to correct misinformation that could affect their choices.^27^ For example, patients may have misconceptions regarding the ease of reversal of permanent contraceptive methods that providers should address. Obtaining true informed consent depends on clinicians' ability to explain the risks and benefits of all appropriate methods of contraception while promoting open dialogue with patients about their preferences, understanding of available methods, and plans for future reproduction.

**Table 1.** Toward a Patient-Centered Shared Decision-Making Approach: Recommendations for Sterilization Counseling

Initiating the conversation
(1) Elicit patient values and reproductive goals by asking open-ended questions.
(2) Discuss which factors are most important to the patient in terms of contraceptive decision-making (e.g., efficacy, ease of use, lack of hormones, noncontraceptive benefits such as lighter menses or acne reduction, privacy, cost, and side effects).
(3) Examine personal biases toward the patient based on age, race, socioeconomic status, etc.
Educating and correcting misinformation
(1) Educate patients about all relevant contraceptive methods and associated risks based on stated values and preferences.
(2) Utilize a multimodal approach to counseling when appropriate, including images, models, and written materials.
(3) Discuss the relative efficacy of various options while recognizing that patients often base contraceptive decision-making on factors other than efficacy.
(4) When discussing LARC as an alternative to sterilization, shift emphasis away from reversibility and instead discuss comparative efficacy, potential noncontraceptive benefits, and ease of initiation relative to sterilization.
(5) Assess the patient's risk of acquiring sexually transmitted infections and discuss benefits of dual protection with a barrier method.
Obtaining informed consent
(1) Provide complete information about the procedure and associated risks.
(2) Emphasize relative permanence of sterilization while still demonstrating respect for patient autonomy and preference.
(3) Invite patients to process the information discussed and return for a follow-up visit if desired, and offer contraceptive options for use in the interim if needed.
Recognizing and minimizing structural barriers to consent and care
(1) Ensure Medicaid sterilization consent forms are signed sufficiently early before delivery to be valid and available during the inpatient postpartum period.
(2) Champion inpatient postpartum LARC placement as well as inpatient and interval postpartum sterilization programs.
(3) Advocate against the sterilization of incarcerated women.
(4) Implement standardized contraceptive counseling programs to obviate the impact of implicit bias.

LARC, long-acting reversible contraception.

On a systemic level, we urge the reframing of contraceptive counseling away from tiered effectiveness-based charts. Labeling permanent contraceptive methods and LARC as “tier one” solely prioritizes efficacy, disregarding the complexity of contraceptive decision-making and the fact that significant variation exists in conceptions of pregnancy intendedness and desirability.^15^ In addition, clinician and institutional performance evaluations that utilize numbers of patients choosing permanent contraception or LARC are inappropriate, as they prioritize public health goals over individual patient preferences. Thus, language such as “success” or “failure” is used to describe whether client selects a certain method that is both ethically inappropriate and also does not clinically represent the complexity of contraceptive decision-making. Supporting patients' preferences through shared decision-making can offset mistrust of the medical system, rebuild the patient–provider relationship that has been undermined by past coercive policies and contemporary bias, and safeguard against the continued impact of structural racism and implicit bias.

## Disclaimer

This article is solely the responsibility of the authors and does not represent the official views of the NIH, the MetroHealth System, or the University of Colorado.

## Abbreviation Used

LARC: long-acting reversible contraception
